# Papillary intralymphatic angioendothelioma: Dabska tumor^☆^^☆☆^

**DOI:** 10.1016/j.abd.2019.03.008

**Published:** 2020-02-12

**Authors:** Thadeu Santos Silva, Luciana Rebouças de Araujo, Geise Rezende Paiva, Rodrigo Guimarães Andrade

**Affiliations:** a Dermatology Clinic, Escola Bahiana de Medicina e Saúde Pública, Universidade Federal da Bahia, Salvador, BA, Brazil; b Pathological Anatomy Service, Studart & Studart Laboratory, Salvador, BA, Brazil

**Keywords:** Histology, Lymphatic vessel tumors, Neoplasms, Soft tissue neoplasms, Vascular tissue

## Abstract

Papillary intralymphatic angioendothelioma (Dabska tumor) is a rarely metastasizing lymphatic vascular neoplasm that usually affects children and young adults. The majority of these cases occur in soft tissues of extremities, and to date less than 40 cases have been described. Despite the generally indolent evolution, can be locally invasive with the potential to metastasize. We describe a case of a young woman presenting with a plantar lesion, for 9 months and histological diagnosis of Dabska tumor. This neoplasm should be considered in the differential diagnosis of vascular dermatoses, allowing early diagnosis and treatment. Long-term follow-up should be performed.

A 35-year-old female patient sought a dermatologist, complaining of an injury to the sole of the right foot 9 months prior. She reported feeling only a slight size increase. Upon clinical examination, there was an area of violaceous staining, with poorly defined limits, on the sole of the right foot (Fig. 1). Dermoscopy showed a homogeneous global pattern of violet color, with pigment distribution in furrows and ridges, as well as dark red spots inside the lesion (Fig. 2). An incisional biopsy was performed, whose anatomopathological study evidenced an intralymphatic lesion, with prominent papillary formations and vascular axes lined by rounded and hyperchromatic cells, projecting into the lumen (hobnail cells), compatible with papillary intralymphatic angioendothelioma – Dabska tumor (Figure 3, Figure 4). Wide surgical excision was indicated and the patient continues to be monitored, with no sign of metastasis.

**Figure 1:**
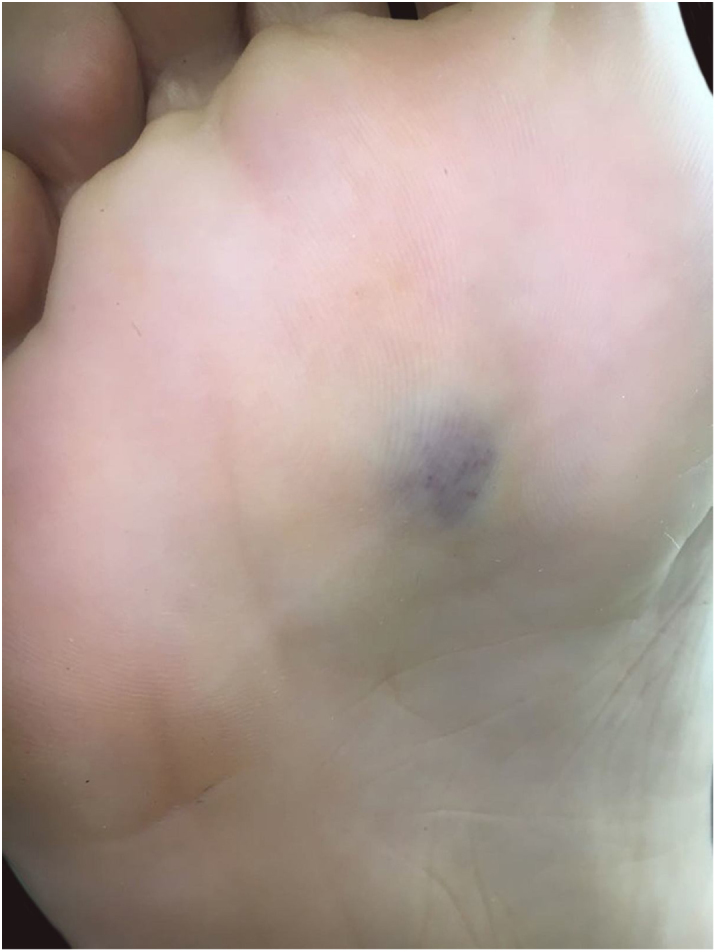
Violaceous lesion, with poorly defined limits, on the sole of the right foot.

**Figure 2:**
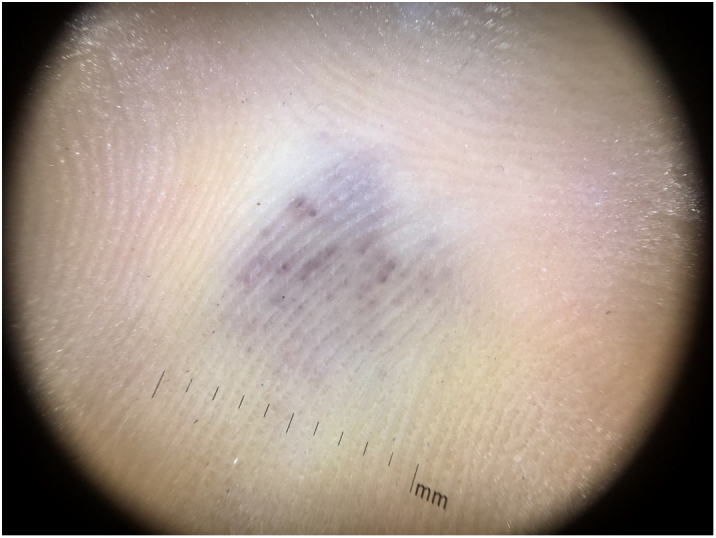
Dermoscopy showed homogeneous global pattern of violaceous staining with blackened red dots, distributed inside the lesion.

**Figure 3:**
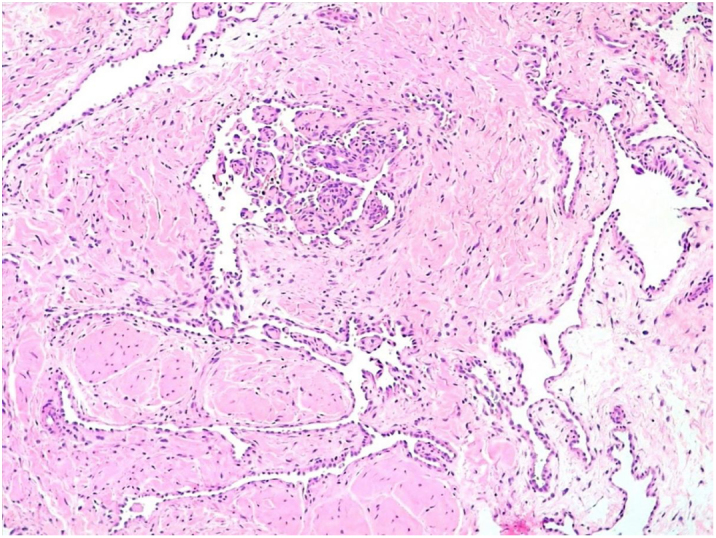
Histopathology. Papillary projections with vascular axes lined by rounded and hyperchromatic cells – “Hobnail cells” (Hematoxylin & eosin, ×100).

**Figure 4:**
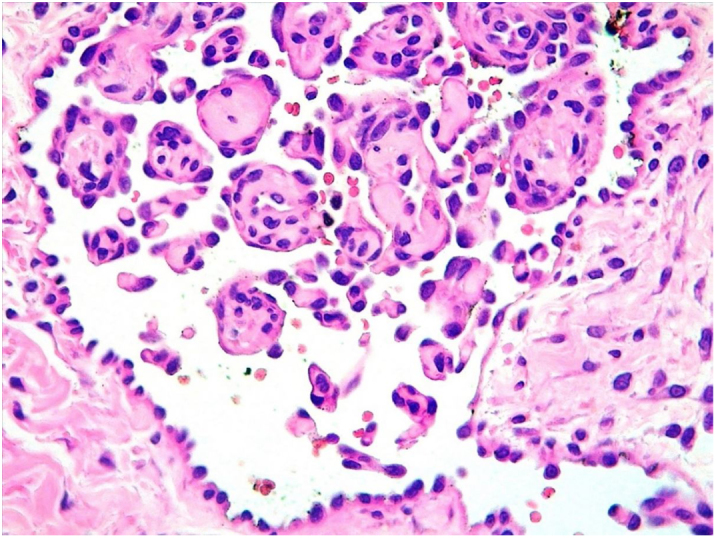
Histopathology. Higher magnification with rounded and hyperchromatic cells that are protruding into the lumen – “hobnail cells” (Hematoxylin & eosin, ×400).

Papillary intralymphatic angioendothelioma (PILA or Dabska tumor) is a rare vascular neoplasm, defined as low grade malignancy tumor, with high tendency of local recurrence.^1^, ^2^, ^3^ Originally considered a malignant tumor and called malignant endovascular papillary hemangioendothelioma, it was renamed in 1998, due to its borderline behavior, prominent presence of lymphatic vessels and the presence of intravascular papillary proliferation.^2^ To date, less than 40 cases have been reported in the literature, mostly affecting soft tissue.^2^ Affects mainly children and young adults, and may be present at birth, without predilection for sex.^2^ Its diagnosis is often a challenge for the pathologist due to its rarity, multifocality and morphological characteristics.^2^ Clinically, the lesion may present as an intradermal nodule or discrete superficial nodule, with slow growth, purplish, pink or bluish coloration and large variation in size (up to 40 cm).^3^ In some cases, it may present with superficial ulceration and/or satellite nodules.^2^, ^3^ It is most commonly located in the dermis and subcutaneous cellular tissue of the extremities, and can also affect the trunk, head and neck, with rare cases described in deeper locations – such as spleen, tongue, testis and bones.^2^, ^3^, ^4^, ^5^, ^6^ Histopathologically, the tumor is characterized by presenting, in the dermis and/or subcutaneous tissue, thin-walled intercomposite vessels lined by endothelial hobnail cells, forming the characteristic intraluminal papillary projections, which assume a focal pattern in rosettes or “match-head”.^2^, ^3^, ^7^ Glomeruli-like structures may be present.^2^, ^3^, ^7^ Mitoses are rare and necrosis is absent.^7^, ^8^ The immunohistochemical study demonstrates positivity for VEGFR-3 and podoplanin (D2-40) in the hobnail endothelial proliferations, indicative of lymphatic differentiation.^8^, ^9^, ^10^ The differential diagnosis is made with reactive angioendotheliomatosis, benign intravascular endothelial hyperplasia and retiform hemangioendothelioma, which present negative immunohistochemistry for podoplanin (D2-40).^3^, ^9^ It has a generally indolent development, however, it may be locally invasive, with rare reports of dissemination to regional lymph nodes and even distal metastases.^3^ The gold standard treatment is wide surgical excision with free margins, which presents an excellent prognosis.^2^, ^3^, ^7^ Thus, its recognition by the dermatologist becomes important to determine early diagnosis and treatment. Long-term clinical follow-up of these patients is mandatory.^2^, ^3^, ^8^, ^9^, ^10^

## Financial support

None declared.

## Authors’ contributions

Thadeu Santos Silva: Approval of the final version of the manuscript; conception and planning of the study; obtaining, analysis, and interpretation of the data; intellectual participation in the propaedeutic and/or therapeutic conduct of the studied cases; critical review of the literature.

Luciana Rebouças de Araujo: Approval of the final version of the manuscript; elaboration and writing of the manuscript; obtaining, analysis, and interpretation of the data; critical review of the literature; critical review of the manuscript.

Geise Rezende Paiva: Approval of the final version of the manuscript; conception and planning of the study; effective participation in research orientation; intellectual participation in the propaedeutic and/or therapeutic conduct of the studied cases; critical review of the literature.

Rodrigo Guimarães Andrade: Approval of the final version of the manuscript; conception and planning of the study; obtaining, analysis, and interpretation of the data.

## Conflicts of interest

None declared.
